# Propensity to trust shapes perceptions of comforting touch between trustworthy human and robot partners

**DOI:** 10.1038/s41598-024-57582-1

**Published:** 2024-03-21

**Authors:** Irene Valori, Yichen Fan, Merel M. Jung, Merle T. Fairhurst

**Affiliations:** 1 Chair of Acoustics and Haptics, Technische Universität Dresden, Dresden, Germany; 2 Centre for Tactile Internet with Human-in-the-Loop (CeTI), Technische Universität Dresden, Dresden, Germany; 3 Chair of Industrial Design Engineering, Technische Universität Dresden, Dresden, Germany; 4 6G-Life, Dresden, Germany; 5 Department of Cognitive Science and Artificial Intelligence, Tilburg University, Tilburg, The Netherlands

**Keywords:** Human behaviour, Psychology

## Abstract

Touching a friend to comfort or be comforted is a common prosocial behaviour, firmly based in mutual trust. Emphasising the interactive nature of trust and touch, we suggest that vulnerability, reciprocity and individual differences shape trust and perceptions of touch. We further investigate whether these elements also apply to companion robots. Participants (n = 152) were exposed to four comics depicting human–human or human–robot exchanges. Across conditions, one character was sad, the other initiated touch to comfort them, and the touchee reciprocated the touch. Participants first rated trustworthiness of a certain character (human or robot in a vulnerable or comforting role), then evaluated the two touch phases (initiation and reciprocity) in terms of interaction realism, touch appropriateness and pleasantness, affective state (valence and arousal) attributed to the characters. Results support an interactive account of trust and touch, with humans being equally trustworthy when comforting or showing vulnerability, and reciprocity of touch buffering sadness. Although these phenomena seem unique to humans, propensity to trust technology reduces the gap between how humans and robots are perceived. Two distinct trust systems emerge: one for human interactions and another for social technologies, both necessitating trust as a fundamental prerequisite for meaningful physical contact.

## Introduction

When we feel vulnerable or distressed, we will often reach out to touch or be touched for comfort. The toucher’s brain activates empathy and reward related regions^1–4^ and the touchee’s distress is reduced at both self-reported and neurophysiological levels^5^, as touch facilitates emotion regulation^6^. On the other hand, bodily contact poses inherent risks by reducing self-other boundaries (e.g., COVID-19 social distancing to avoid disease spread). Mutual trust may be a key prerequisite for people to get physically in touch with one another. The COVID-19 pandemic showed us the negative effects of social isolation, particularly on the lack of physical contact with our loved ones^7,8^. It also showed us the dramatic effects of the shortage of medical and health personnel to care for the sick. In this apocalyptic scenario, technologies have attracted particular interest in our society, because of their potential to connect us with others and make basic services such as health and education accessible to geographic areas and population groups that are difficult to reach by local services. Specifically, robots have been looked at as tools with very high potential for support in various functional and affective domains^9^. When it comes to care robots that can engage in social exchanges, to touch and be touched in affective ways, many questions arise. Under which conditions can we perceive them as trustworthy partners? How does it feel to be touched and comforted by a robot? Are there individual differences with regard to each person's propensity to trust technology?

According to traditional definitions, trust is the willingness to be vulnerable to the actions of another party based on positive expectations about the trustee’s behaviour^10^. Notably, a state of trust is associated with reduced physiological arousal^11^ and cognitive monitoring^12^. There are several dimensions of trust. Firstly, people have a general predisposition to trust others^13^, which we can call propensity to trust. This dispositional aspect of trust is plastic, varying a lot from person to person and also changing across one’s lifespan and with experience. Secondly, situational trust relies on the specific characteristics of the trustee (ability, integrity, benevolence), and is context-dependent^14^. We can more precisely refer to perceived trustworthiness when we delve into the characteristics that make a trustee more or less trustworthy. Some authors suggest that we can further distinguish between cognitive and affective dimensions of trust and perceived trustworthiness^15^. They propose that one’s ability and integrity are strong predictors of cognition-based trustworthiness (expectations grounded in cost–benefit economic reasoning), whereas benevolence and values congruence are stronger predictors of affect-based trustworthiness (built upon positive affective bond between the parties).

It's interesting to note that most studies on trust investigate organisational contexts, work relationships and marketing, which inherently might draw more upon cognitive dimensions of trust. We know much less about the foundations of interpersonal trust and perceived trustworthiness in affective contexts where the sharing of emotions, vulnerability, empathy, and mutual support form the true cornerstone of interactions and relationships. For instance, friends may be perceived as trustworthy because of their benevolence, whereas leaders are trusted because of their integrit^16^. Behavioural paradigms for measuring trust and trustworthiness have certainly favoured the cognitive and socio-economic interpretation of trust. In the widely used Trust Game, 2 individuals are assigned the roles of investor and trustee. The investor possesses a designated sum of money and has the option to transfer a portion to the trustee. Upon doing so, the transferred sum is tripled, and the trustee can reciprocate by sending back an amount to the investor. The sum of money the investor chooses to transfer to the trustee serves as an indicator of their willingness to trust the recipient, while the sum returned by the recipient reflects their trustworthines^17^.

Notably, most of the time researchers conceive the trustor as the vulnerable party that depends upon a “strong” trustee. Things in everyday relationships are more complicated than that and we may trust someone not only because they are able to help and comfort us when we are not self-sufficient, but also because they are willing to open up and share their own vulnerabilities. Some authors found that seeing someone in tears can improve trust, resulting in more money given to them as the receiver of a Trust Game^18^. Several socio-affective mechanisms and cues drive interpersonal trust and perceived trustworthiness^19^. A long tradition of studies has focused on the presentation of sequences of neutral and decontextualized faces, suggesting that people automatically judge faces on a trustworthiness dimension^20^, with a certain level of agreement among observers, particularly for faces belonging to social in-groups^21^. However, what these approaches overlook is the relational nature of trust and perceived trustworthiness, as well as the crucial role of context. Previous research has especially neglected the role that trust may play in shaping attitudes and behaviours related to social touch. Being willing to trust others and perceiving the partner of a specific interaction as trustworthy may facilitate experiencing a tactile exchange as appropriate and pleasant, especially in situations of emotional vulnerability.

We live in an increasingly technological world, yet we know so little about the cognitive and behavioural mechanisms that shape our interactions with technology. Are we ready to (should we) transfer the mechanisms that govern human interactions to those mediated by technology? In what contexts, under which conditions, and for what purposes can we trust social robots? The very concept of what constitutes a robot can be subject to lengthy debates. Robots are tools designed with specific features and affordances, which determine the ways in which they can be interacted with and their function. In the case of certain types of robots, their primary function is to act as social entities capable of communication and forming relationships. Companion robots, also known as social or assistive robots, are programmed to interact and engage with humans in social settings, providing assistance, companionship, and emotional support (for a review on definitions, see^22^). Companion robots find applications in a wide range of settings, including healthcare and education, especially for older people and children. In healthcare, several types of robots have been integrated in care services^23^, but evidence of effectiveness is often limited^24^. In education, companion robots have been utilised to enhance learning experiences^25^. When a social robot was integrated into a toddler classroom for over five months, the interaction quality between children and the robot improved over time, becoming more and more similar to peer-to-peer relationships^26^. However, also in this research field and applicative context, there is a lack of solid evidence in support of the specific benefits that robots can bring to children’s lives.

To use companion robots for meaningful interactions, it is crucial to leverage the cognitive and behavioural mechanisms underlying effective social interactions, delving into how these mechanisms operate with technology. For people to be willing to interact with any specific technology, trust is key^27^. Within the context of the Trust Game, the amount of money transferred by the investor is influenced by the investor's perception of interacting with a human rather than a computer^28^. A machine’s trustworthiness in terms of ability, benevolence, and integrity increases as the technology is perceived to be more human-like and personified. Conversely, trustworthiness related to system attributes (functionality, usefulness, reliability) hold more relevance for technologies perceived as object-like^29^. The framework known as Computers as Social Actors (CASA) proposes that machines can possess personalities, prompting people to interact with them as if they were human, even while being aware of their non-human nature^30^. This goes beyond mere anthropomorphism, as the acceptance and trust in Artificial Intelligence (AI) technologies also require empathy, leading to informative, enjoyable, effortless, and person-centred interactions^31^.

We have discussed how social touch is particularly intriguing as a means for connecting with others, comforting and being comforted. In clinical applications, the possibility to give robots the superpower of social touch is of particular interest to leverage the many therapeutic effects of touch. For instance, pet-like robots that can be interacted with on a tactile level have been found to improve the well-being of people with dementia^32^ and facilitate anxiety management^33^. In education, a social robot has been integrated in a toddler classroom for months, and children's tactile behaviours were particularly informative of the relationship with the robot. Specifically, children touched the robot quantitatively and qualitatively differently from the way they touched a similar inanimate robot or soft toy^26^. Since social and affective touch can help children in social^34^ and cognitive^35^ learning processes, more research is needed to unveil the potential (and limits) of using social robots that can engage in touch interactions with children.

Yet, there is evidence suggesting that touch from a robot is not perceived to be as pleasant as touch from a human, sometimes failing to elicit the same neurophysiological and affective reactions^36,37^. We therefore need to better understand which aspects of human–robot social tactile interaction influence its meaning and effects. Firstly, individual differences play a big role in how human–robot touch is perceived. Observers with negative attitudes towards robots, in general, are more likely to perceive a robot touching a human as machine-like rather than human-like^38^. Moreover, several situational characteristics related to the context in which the interaction takes place and how it feels can modulate social touch perception. For instance, the role each partner plays in terms of sender or receiver is key^39^. Pet-like robots for therapy have been mainly conceived as touch receivers^32,33^. A touch-receiving social robot has been found to reduce pain perception and increase mood in human touchers^40^. In other works social robots were tested as touch senders, with robot touch being associated with reduced physiological stress^41^ and increased compliance with a request^42^. Additionally, social touch can be initiated, reciprocated, and become a dynamic, two-way communicative exchange. In a study, participants watched videos featuring a small humanoid robot assisting a human with computer issues. The agents either never touched or engaged in robot-initiated, human-initiated, or reciprocal touch. The robot's proactiveness was manipulated, with it offering help on its own initiative (proactive) or upon request (reactive). Observers perceived the robot as less machine-like when it was proactive and more machine-like when it was reactive, highlighting initiation as a fundamental ability of humans and human-like machines^38^. Additionally, using a human-sized teddy bear robot, researchers found that reciprocity of touch is key, with hugs reciprocated by the robot vs only initiated by the human leading to longer exchanges and higher self-disclosure from the participant^43^, as well as increasing prosocial behaviours^44^. However, it is not yet clear whether a social robot is perceived as more or less trustworthy depending on the type of role it assumes (sender or receiver) and the type of touch (human-initiated or robot-initiated, with or without reciprocity). Similarly, individual dispositional characteristics related to attitudes towards social touch have enormous effects on how different users may perceive and use touch with robots^45^.

On the issue of what situational and dispositional factors influence the effects of human–robot social touch, the concept of trust is finding particular interest due to its dual nature (situational and dispositional) and the plasticity with which it can change with experience and be tuned. Trust in a robot is influenced by many individual human factors and dispositions such as demographics, personality traits, attitudes and implicit cognitive mechanisms^46^. Most research on the situational factors modulating the trust-touch link investigated whether social touch has the power of boosting trust in human–human or human–robot interactions (for a systematic review, see^47^). Provided that the robot-initiated touch is perceived as appropriate (which is not always the case), it has been shown to promote the robot’s perceived trustworthiness^48^. Touch from a robot providing feedback to a person facing a computer issue increased observers' perception of the robot's trustworthiness on both functional and personal dimensions^49^, especially when the robot was more human-like^50^. During 1st-person interaction with a robot providing assistance on a specific task, older adults reported higher trust if the robot-initiated touch compared to no touch conditions, but only if the robot did not make mistakes^51^. Using a human–robot Ultimatum game, researchers found that touch from a robot can buffer individuals’ reactions to the robot’s unfair actions^52^. In another study, holding hands with a robot while watching a scary video led to higher trust when the robot’s hand was warm compared to cold or in no touch conditions^53^. Overall, social “touch does not increase prosocial behaviours in the absence of meaningful social and psychological connotations”^54^. On the other hand, it is rarely investigated whether trust is an essential prerequisite for people to have positive perceptions of human–robot touch and be willing to interact with robots using touch. Our research is situated within this gap in the literature.

### The present study

Previous accounts have proposed that touching enhances prosocial behaviours but here we test whether trust promotes positive appraisal of social touch. We ask how people perceive observed interactions between humans or a human and a companion robot that engage in tactile exchanges with a comforting intent. We aim at investigating if people would trust a person or robot when they offer comfort to a vulnerable other, or rather they express vulnerability and receive comfort. We investigate what specific role different dimensions of situational trust (i.e., perceived trustworthiness across the dimensions of ability, benevolence, and integrity) play in people’s perceptions of the different scenarios. Moreover, we explore how the interactions are perceived in terms of realism, appropriateness and pleasantness of the touch, and affective state (valence and arousal) associated with the exchange. Lastly, we question whether people’s perceptions are moderated by individual dispositional characteristics such as propensity to trust other people and technology in general, and attitudes toward interpersonal touch.

**Figure 1:**
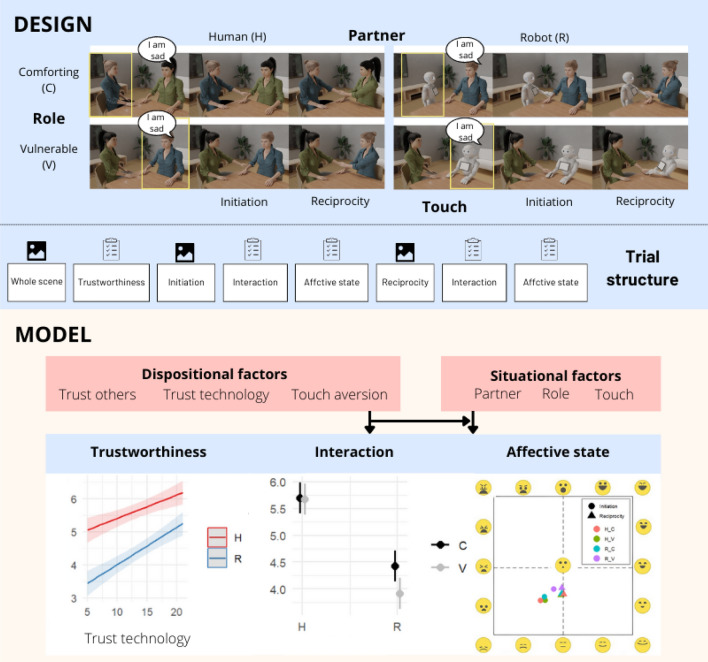
**Experimental design and theoretical model.** The design panel depicts experimental conditions resulting from the Partner (human, robot) * Role (comforting, vulnerable) combinations. Each of the 4 resulting scenes include 2 touch phases (initiation, reciprocity). The yellow circle indicates which character is the target of the questions on perceived trustworthiness. The trial structure consists of (i) presentation of the whole scene followed by trustworthiness questions, (ii) presentation of the touch initiation segment of the scene followed by questions on how the interaction is perceived (how realistic the exchange, how appropriate and pleasant the touch), (iii) presentation of the touch reciprocity segment of the scene followed by questions on how the interaction is perceived. The model panel visualises the hypothesised effects of dispositional and situational factors on the dependent variables. The graphs present a summary of the main results.

We hypothesise that certain factors modulate (and perhaps increase) the perceived trustworthiness of a character. Since social touch is an intimate contact that brings us closer to each other's vulnerabilities, a character might be perceived as trustworthy if they are capable not only of being comforting but also of showing vulnerability themselves. When it comes to interactions that focus on affective content and involve social touch, we expect people to perceive other humans as more trustworthy than robots. However, we wonder whether the ability to express vulnerability would act as a humanising factor and improve the robot’s perceived trustworthiness. We also hypothesise that reciprocity (i.e., observing the touchee touching the toucher back) induces more positive perceptions of the tactile interaction compared to when touch is unidirectional. Whether this is also the case in human–robot interactions will be explored. Lastly, we expect people’s perceptions to be moderated by individual differences, with propensity to trust others being positively associated with perceptions of human–human interactions, propensity to trust technology positively associated with perceptions of human–robot interactions, and touch aversion generally leading to less positive evaluations of observed social touch. Figure 1 depicts the experimental design and theoretical model.

## Results

### Trustworthiness

**Figure 2:**
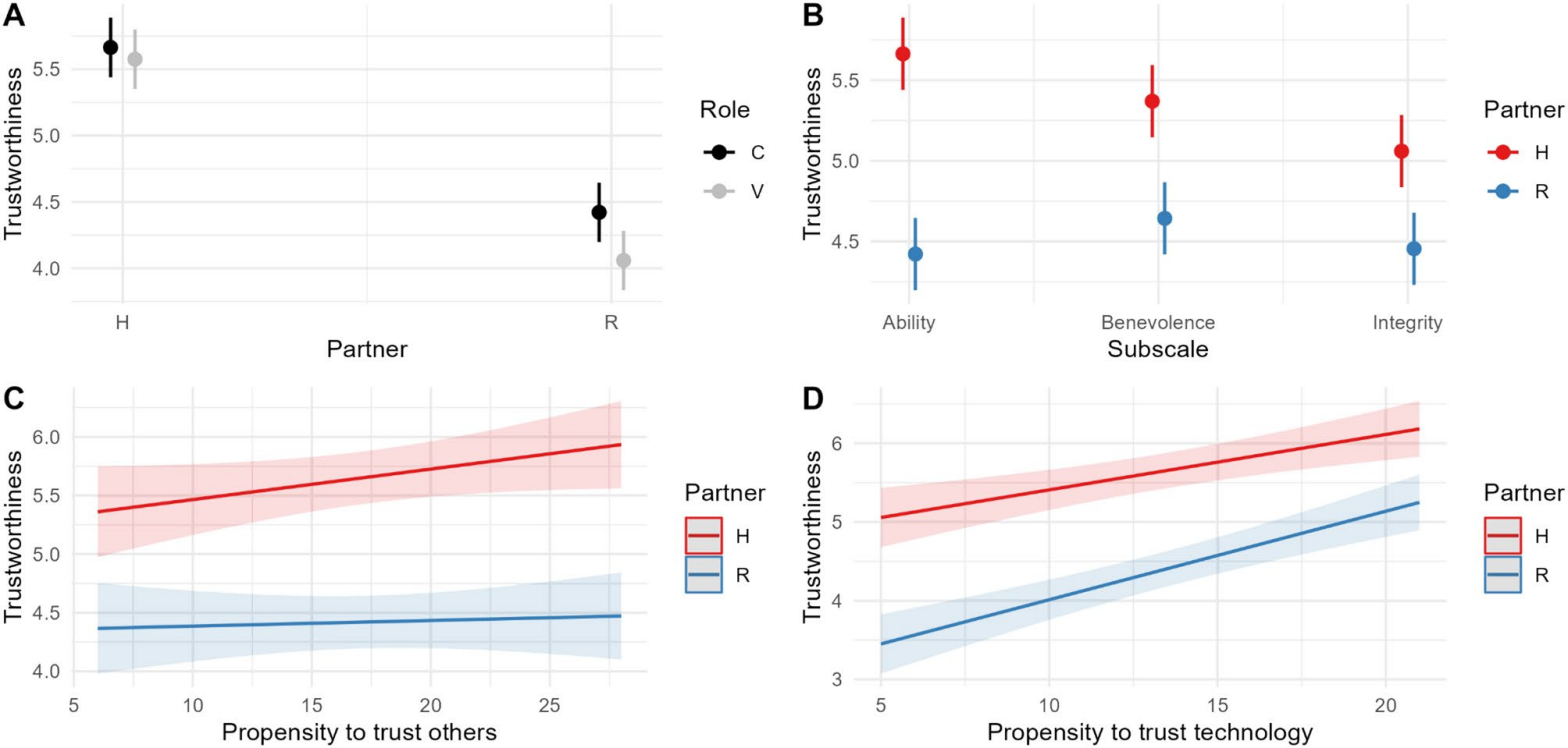
**The interactive nature of trust.** Significant effects predicted by the model on perceived Trustworthiness; n_participants_ = 152; n_observations_ = 1824. The results indicate that humans are equally trustworthy when comforting the other or expressing vulnerability (**A**). The robot is perceived as less trustworthy than humans, especially when it expresses vulnerability (**A**). What makes humans more trustworthy is specifically the ability dimension of trust, which emerges as the most impactful gap between humans and the robot (**B**). Individual differences in participants' propensity to trust moderate these effects. While the propensity to trust others increases the perceived trustworthiness of human characters in the observed interaction (**C**), the propensity to trust technology is positively associated with the perceived trustworthiness of the robot (**D**), reducing the gap between humans and the robot.

The model includes the significant effects of Partner * Role (χ2 = 8.5, df = 1, p = 0.004), Partner * Subscale (χ2 = 34.5, df = 2, p < 0.001), Partner * Propensity to trust others (χ2 = 5.5, df = 1, p = 0.02), Partner * Propensity to trust technology (χ2 = 11.6, df = 1, p < 0.001). Indices of the model goodness of fit are: R2 (fixed effects) = 0.23; R2 (total) = 0.51. Figure 2 visualises the significant effects predicted by the model.

### Interaction

#### Interaction realism

The model includes the significant effect of Partner * Role (χ2 = 21.02, df = 1, p < 0.001), Propensity to trust others (χ2 = 5.86, df = 1, p = 0.02), Partner * Propensity to trust technology (χ2 = 16.26, df = 1, p < 0.001). Indices of the model goodness of fit are: R2 (fixed effects) = 0.31; R2 (total) = 0.60. Figure S1 in the Supplementary Information visualises the significant effects predicted by the model. The results suggest that human-to-human interactions are perceived as more realistic than human–robot interactions. The latter is less realistic, especially when the robot expresses vulnerability. Individual differences among participants moderate these effects. In general, social interactions involving comforting touch are perceived as more realistic by those who trust others more. The propensity to trust technology is linked to perceptions of human–robot interactions as more realistic.

### Touch appropriateness

**Figure 3:**
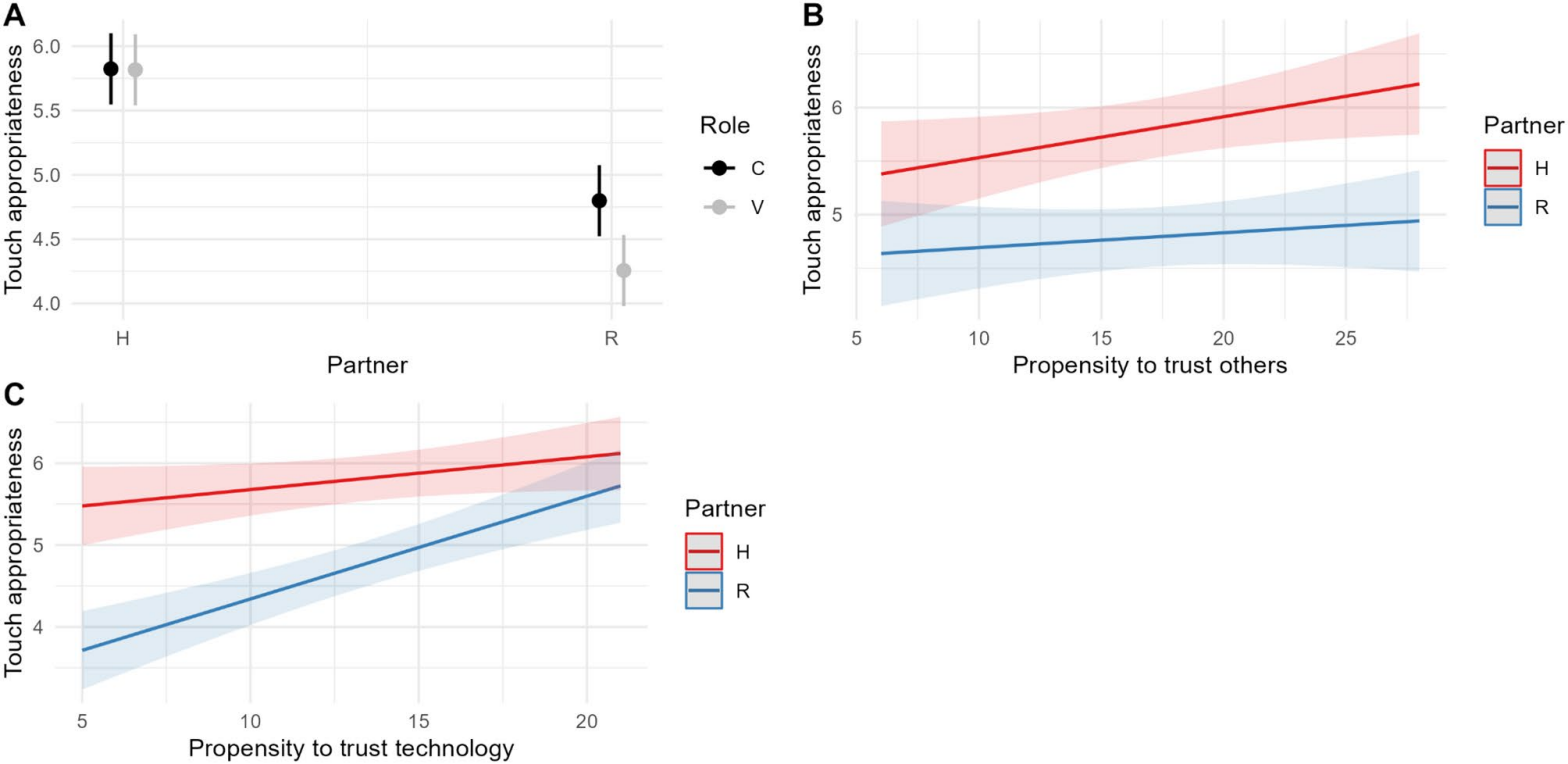
**Trust promotes positive appraisal of social touch.** Significant effects predicted by the model on Touch appropriateness; n_participants_ = 152; n_observations_ = 1216. Human-to-human comforting touch is perceived as more appropriate than human–robot touch. The latter is less appropriate, especially when the robot expresses vulnerability (**A**). While the propensity to trust others increases the perceived appropriateness of human-to-human touch (**B**), the propensity to trust technology is positively associated with the perceived appropriateness of the robot touch (**C**).

The model includes the significant effect of Partner * Role (χ2 = 19.96, df = 1, p < 0.001), Partner * Propensity to trust others (χ2 = 4.43, df = 1, p = 0.04), Partner * Propensity to trust technology (χ2 = 29.52, df = 1, p < 0.001). Indices of the model goodness of fit are: R2 (fixed effects) = 0.24; R2 (total) = 0.59. Figure 3 visualises the significant effects predicted by the model.

### Touch pleasantness

The model includes the significant effect of Partner * Role (χ2 = 15.65, df = 1, p < 0.001), Propensity to trust others (χ2 = 3.91, df = 1, p = 0.05), Partner * Propensity to trust technology (χ2 = 39.68, df = 1, p < 0.001). Indices of the model goodness of fit are: R2 (fixed effects) = 0.26; R2 (total) = 0.62. Figure S2 in Supplementary Information visualises the significant effects predicted by the model. Results show that human-to-human comforting touch is perceived as more pleasant than human–robot touch. The latter is less pleasant, especially when the robot expresses vulnerability. Individuals’ propensity to trust others is associated with increased pleasantness. Moreover, individuals’ propensity to trust technology is associated with increased perception of robot touch as pleasant, thus reducing the gap between humans and robots.

### Characters’ affective state

**Figure 4:**
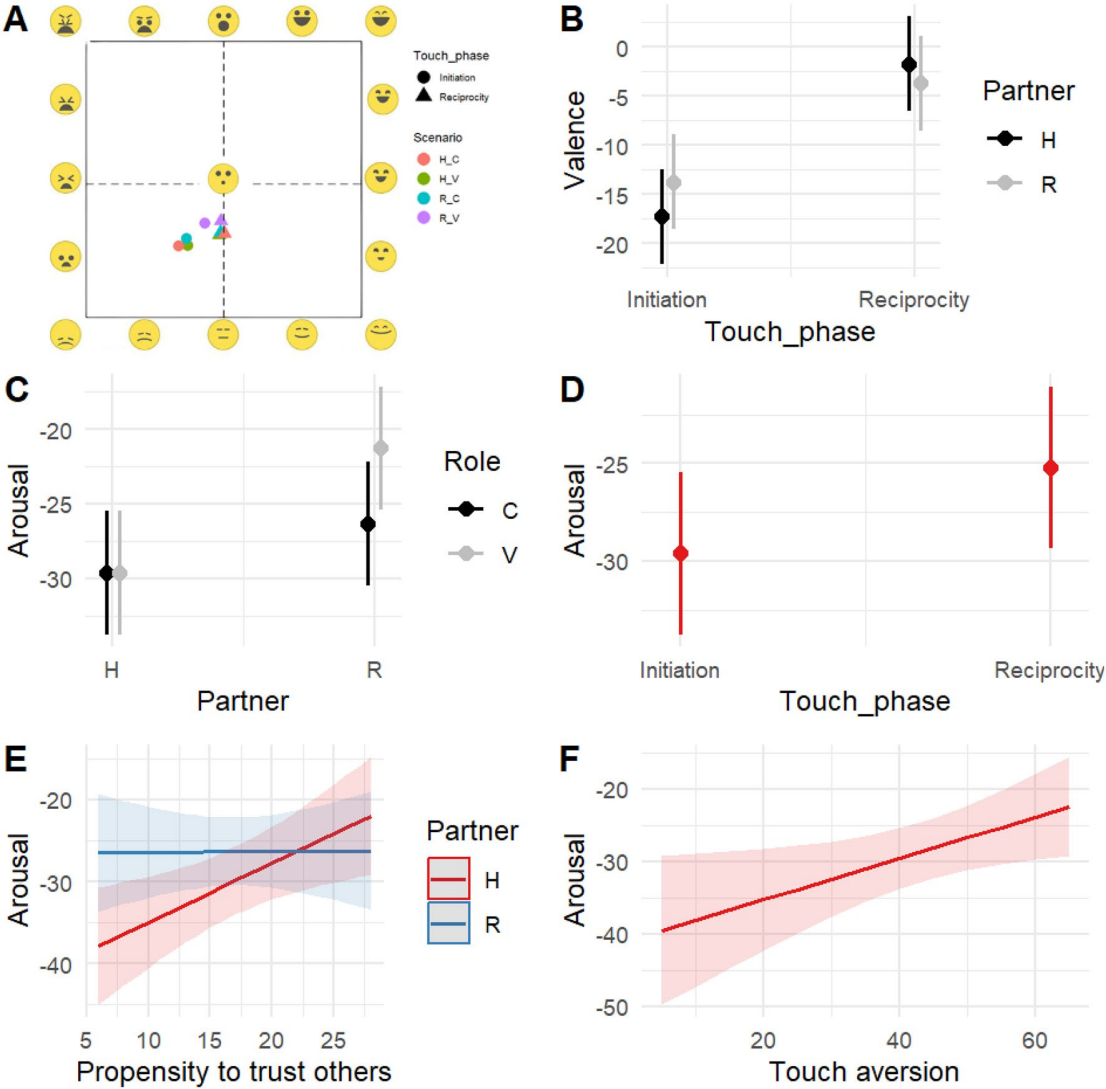
**How does it feel?** Significant effects predicted by Valence and Arousal models; n_participants_ = 142; n_observations_ = 1115. Panel (**A**) visualises participants’ clicks on the EmojiGrid, averaged by experimental condition (scenario) and touch phase. Results suggest that the characters of the observed interaction are perceived to be in a less negative affective state when touch is reciprocated by the receiver. That effect is moderated by the Partner factor, with the reciprocity effect being smaller in the Robot condition (**B**). A more neutral arousal state (closer to 0, which represents the center of the EmojiGrid) is reported when touch happens between a human and the robot rather than two humans. This is especially evident when the robot is the one expressing vulnerability (**C**). Arousal also becomes more neutral when touch is reciprocated by the receiver, with no significant difference between human and robot partner (**D**). Individuals’ propensity to trust others is associated with more neutral perceived arousal, only in human-to-human interactions (**E**). Individuals’ touch aversion is associated with more neutral arousal (**F**).

Since these data are derived from spatial coordinates dependent on the size of the viewing window on each participant's screen, we have retained the data from participants who maintained this window at a constant size throughout the task. Six participants whose screen dimensions changed during the task (e.g., they resized the experiment platform window) were excluded from these analyses. Moreover, 4 participants did not provide a valid response to these questions (i.e., did not click on a point inside the EmojiGrid). Therefore, these analyses are based on n_participants_ = 142 and n_observations_ = 1115. Figure 4 visualises the significant effects predicted by the models on valence and arousal.

As for valence, the model includes the significant effect of Partner * Touch phase (χ2 = 7.41, df = 1, p = 0.006). Indices of the model goodness of fit are: R2 (fixed effects) = 0.08; R2 (total) = 0.53. As for arousal, the model includes the significant effects of Partner * Role (χ2 = 9.27, df = 1, p = 0.002), touch phase (χ2 = 13.99, df = 1, p < 0.001), Partner * Propensity to trust others (χ2 = 17.72, df = 1, p < 0.001), touch aversion (χ2 = 5.06, df = 1, p = 0.02). Indices of the model goodness of fit are: R2 (fixed effects) = 0.07; R2 (total) = 0.55.

## Discussion

In this study, adult participants from around the world observed and evaluated scenes of social interactions between two humans or a human and a robot, described as peer familiar relationships. In different experimental conditions, one of the characters expressed emotional vulnerability by saying, "I am sad," and the other performed a comforting gesture by touching their arm. In response to this gesture, the character who was touched reciprocated the touch. Participants were asked to assess how trustworthy the character providing or receiving comfort was in terms of ability, benevolence, and integrity. Additionally, observers rated the realism of the interaction, the appropriateness and pleasantness of the touch, the valence and arousal attributed to characters', distinguishing between phases where touch was initiated and reciprocated. We support the idea that trust is an interpersonal bond amplified by our paradigm which uses a social touch exchange to emphasise the interactive nature of the scene. We show that trust promotes positive appraisal of social touch, with the experimental manipulations of the social scenario, mediated by observers’ propensity to trust, resulting in differences in perceived trustworthiness, perceptions of the interaction and associated affective states. In addition, we shed light on the limitations of applying these concepts to companion robots. Nevertheless, propensity to trust is a subjective and potentially plastic trait that can be leveraged to facilitate acceptance of technologies through positive experience. Showing that if we trust, then social touch will be perceived as more appropriate and pleasant, we take a complementary perspective to previous studies that have investigated the reverse relationship (if we touch, we trust).

First and foremost, we did not find differences in the perceived trustworthiness of individuals based on their role in the interaction. People perceive as equally trustworthy someone who comforts another in a moment of vulnerability and someone who expresses their own vulnerability. This finding significantly expands our understanding of trust, which has been mainly conceived as a one-way perception and behaviour from the trustor to the trustee^10,17^. Trust is rather an interpersonal, interactive mechanism built upon the willingness to share one's vulnerabilities with the other. Observing how these mechanisms operate in human–robot interactions allows us to understand whether they are more or less specific to human interactions, or rather fundamental principles that can be leveraged to build trust in technologies designed for social presence. We found that the robot is overall perceived as less trustworthy than human interaction partners, especially when it expresses vulnerability. This suggests that people may not desire to interact with a social robot that, like a human being, can express vulnerability and receive comfort. Symmetry in human relationships among peers is fundamental for various social processes, including perspective taking and empathy. Instead, we should perhaps consider robots as partners of asymmetric, more unidirectional relationships, where they need to possess specific social skills to provide emotional support to humans. This clearly imposes limitations on the social relationship with a robot and raises important questions about the foundations of human–robot trust, the design and implementation of companion robots. According to previous literature, robots are perceived as less reliable if designed in a more anthropomorphic way^55^. Anthropomorphism of a robot has been found to be implicitly associated with lower agency and capacity to sense and feel compared to humans^56^, potentially because of the mismatch between affordances (what I expect the robot to do given its appearance and features) and actual performance. Indeed, our results indicate that, when comforting one another, humans are perceived as trustworthy especially for their abilities to provide support and assistance to another person, whereas robots are perceived as less skilled for social exchanges. Some promising alternatives for robots that can receive touch and comfort are pet robots, which can be used in healthcare to promote patients’ well-being^32,33,57^. Notably though, perceived trustworthiness of an agent in a specific situation is influenced by observers’ dispositional attitudes, such as their general propensity to trust. Our data suggest that there are two somewhat distinct systems for trusting other people or technology, which specifically come into play in these two different types of (social) interactions.

Secondly, we see that our manipulations of the social scenario, mediated by propensity to trust, results in differences in how the interaction was perceived, with trust promoting positive appraisal of social touch. In human-to-human scenarios, propensity to trust others is positively associated with perceived character’s trustworthiness, interaction realism, touch appropriateness and pleasantness. In human–robot scenarios, ratings of realism, appropriateness and pleasantness are lower. This is especially evident when the robot assumes the vulnerable role. Nevertheless, individual propensity to trust technology reduces the gap between humans and robots. These insights offer a new perspective in the study of the link between touch and trust, where researchers have primarily investigated the role of social touch in promoting and facilitating interpersonal trust, whether mediated or not by technology (see^47^ for a systematic review). Here we look at the other side of this presumably two-way interaction. We propose that trust is a prerequisite for positively perceiving tactile social interactions and that there are two somewhat distinct systems for trusting other people or technology, which specifically influence these two different types of (social) interactions. Additionally, propensity to trust is a subjective and plastic trait with the potential to influence acceptance of technologies through positive experience. It can be hypothesised that with the advancement and widespread use of technology in everyday life, people's overall trust in technologies is likely also to increase. If trust is moderated by familiarity with specific tools^58,59^, we may have to wait for companion robots to appear more regularly in our daily contexts to understand whether future humans will be more inclined to trust and interact with them in affective ways. Studies on the development of trust in children show that familiarity is particularly important in novice learners, and that with increasing social experience, discrimination, e.g., of more or less trustworthy informants, is refined to be increasingly driven by the other’s competence, also when it is a robot^60^. Therefore, trust towards others and robots is plastic and understanding individual differences can aid in personalising robot touch behaviours to optimise interactions.

Lastly, we investigated which affective states are associated with the different social scenarios, particularly in terms of valence and arousal, which are key dimensions for understanding social touch^61,62^. In our paradigm, social touch is used to amplify the interactive nature of a peer-to-peer comforting exchange. We see that reciprocity of touch influences the affective experience, alleviating feelings of sadness (as shown by less negative valence and more neutral arousal). Observers with higher propensity to trust others also attributed more neutral to the characters in the human-to-human scenarios. The power of reciprocal touch and trust is lessened in human–robot interactions, where we see more neutral arousal, especially when the robot assumes the vulnerable role. Previous research found that interpersonal touch is more arousing than object-based touch, suggesting that human-to-human touch is experienced as more intense^62^, and the robot in our study may have been perceived as an object more than a social partner. Such human–robot interaction is therefore perceived as less realistic, appropriate, pleasant, and less emotionally meaningful. We also found that observers with higher aversion towards social touch perceived the scenarios as overall more neutral at the arousal level. If higher touch aversion is associated with higher vigilance to observed social touch (as suggested by the neural responses found by^63^), we could expect the opposite relation between touch aversion and arousal. On the other hand, it is possible that less touchy-feely people are simply less activated by scenarios of vicarious touch, without necessarily showing discomfort or hyper-vigilance. Indeed, valence does not appear to be influenced by individuals’ touch aversion in our data.

It is worth mentioning that this study has some limitations, which open the doors to future research. We focused on the perception of observed social tactile interactions between two humans or a human and a robot. To safeguard the simplicity of experimental design and statistical models, we did not include a control condition in which the interaction did not involve touch. Moreover, we used static pictures instead of animations to avoid confounding aspects such as touch velocity. Comforting touch has well-known optimal velocity ranges in human-to-human interactions^5^. Robots can also be programmed to execute movements with spatio-temporal patterns designed to represent different emotions (e.g., in^64^). However, the movements of real robots are still far from the smoothness of human ones. In general, animating tactile gestures to be nearly realistic but not quite can inadvertently lead observers into the uncanny valley, where the slight discrepancies from reality evoke feelings of unease or discomfort due to the almost-human resemblance without achieving true authenticity.. Moreover, although animations may be more effective than static pictures in facilitating learning, static pictures are more appropriate to illustrate very specific moments of the process (e.g., in our study, we focused on the initiation and reciprocity phases of the comforting interaction)^65,66^. Lastly, it is important to note that in creating human–robot interaction scenarios, we used a specific robot: Pepper, a commercially available humanoid social robot widely used in social touch research^67–69^. We know that the specific physical characteristics (such as anthropomorphism) and functionalities (e.g., facial expressiveness and linguistic production) of a robot have an impact on how it is perceived^70^. Therefore, it is not guaranteed that the results obtained with Pepper are applicable to different types of robots, such as those with higher levels of anthropomorphism^71,72^. This remains an open question to be explored further in future research.

To deepen the role of social touch in human–robot interactions, future studies might not only compare touch and no-touch conditions, but also explore different types of touch. Different combinations of physical parameters of touch, such as velocity, intensity, duration, and contact areas result in different gestures (e.g., stroking, holding, shaking, tapping, squeezing) that convey different emotional meanings, from sadness, to joy, gratitude, and love^73^. This affective haptic vocabulary has been also investigated in human–robot interactions^74^, where it is crucial to disentangle the importance of the robot being able to *understand* and *communicate* through touch. To become a socially intelligent partner a robot must be able to capture and classify human touch and respond to this in an appropriate manner, interpreting not only tactile features but also contextual factors^75^. At the same time, the robot could also be able to touch the human in an affective way, and produce tactile gestures that the human can understand^76^.

The present study is based on an observational task in which participants are exposed to images of social interactions that include touch. Although the participants play the role of simple observers of scenes taking place between two characters, literature suggests that the mere observation of others' touch leads to pleasantness ratings^77^ and brain activity similar to those associated with a first-person experience of touch (e.g., as^78^ found with monkeys). Therefore, the participants' evaluations of the proposed stimuli can be interpreted as an indicator of how they would perceive the social situation themselves. Nonetheless, given that affective tactile interactions with robots are not yet part of our everyday experiences, observational data on this specific social context may not accurately represent the experiences associated with first-hand interactions^79^. Future studies would need to conduct lab-based experiments whereby participants interact with robots. This possibility is challenged by the limited skills and capacity for actual interactivity that robots have at the present time especially with regards to exchanges involving social touch^32,75^. In terms of the possibilities this set-up would open up, among the most fascinating is surely the integration of neural, physiological, and kinematic measurements to characterise human cognition, perception, and action during social interactions with robots.

Although there has been significant progress in creating more advanced and socially adept robots in recent years, there are concerns that the field is entering a winter phase of disillusionment^80^. Researchers are putting a lot of resources into enhancing the naturalness and authenticity of robot behaviours (e.g., designing robots to display emotions and responses that are as realistic as possible), with the idea that this will foster more genuine and meaningful interactions with humans. For instance, robots are being programmed to recognize touch gestures^81^ and to perform touches with optimal sensorimotor features to be perceived as pleasant and non-intrusive^53,82^. However, touch is a communicative signal that takes on various nuances, uses, and interpretations depending on the context and the person giving or receiving it^83^. Our society has yet to establish new social norms for digital social touch, through a dialogue between what is technologically feasible and what is truly desired by and beneficial for the human in the loop^84,85^. It is crucial that we understand under which conditions and in what contexts human–robot interactions can benefit from social touch. To address this, it is essential to clearly define the neurocognitive processes that underpin human–robot interactions, employing neuroscience and psychophysiology techniques to uncover the genuine capabilities and limits of social robots^86^.

In conclusion, perceiving other individuals as trustworthy is crucial in affective exchanges that involve social touch, where barriers between the self and the other are reduced, we share vulnerabilities, offer closeness and comfort. Here we provide evidence that trust is an interpersonal, interactive tango rather than the one-way mechanism from trustor to trustee that has been studied in previous literature. We also show that trust promotes positive appraisal of social touch, offering a complementary perspective to studies that have shown the reverse effect of touch as a trust booster. Looking into the future, we see our lives increasingly intertwined with those of technologies such as robots, which are not only tools but also partners in social exchanges. Yet, we still do not know what social norms apply to these new interactions. The present findings show potential limits to the social power of trust and touch in human–robot interactions, suggesting, however, that leveraging individuals' positive attitudes and trust towards technology can reduce the distance between humans and robots. This will help to shed light on crucial challenges in robot design that we humans could potentially perceive as partners to trust and touch.

## Methods

### Participants

Eligible participants were older than 18 years of age and fluent in English. From the a priori power analysis (see Statistical approach section for details), we aimed at n = 152. We collected data from 153 participants. One participant has been excluded from analyses because they used a mobile device rather than a personal computer, which was required to participate. The final sample is n = 152 (nFemales = 77, nMales = 76; age range = 19:67; meanAge = 29.04; sdAge = 8.81). Despite being given the option to select and specify non-binary gender identities, all participants identified with a female or male gender. Participants come from 33 nationalities across Europe, Africa, America, and Asia. With the intention of representing the general adult population, we did not establish exclusion criteria on the basis of medical or psychological conditions. Self-reported medical or psychological conditions included anxiety and/or depression (n = 9), neurodevelopmental conditions (n = 4) such as ASD (Autism Spectrum Disorder) and/or ADHD (Attention Deficit and Hyperactivity Disorder), medical conditions (n = 5). Participants reported minimal previous experience with robots (mean = 0.68 on a 0 “none” to 4 “a lot” Likert scale). When given the chance to briefly describe such previous experience, only 13 participants mentioned brief, occasional interaction with robots we could call social. These qualitative data are reported in the Supplementary Information.

### Procedure

Participants were recruited via Prolific and compensated 9,63 £ average reward per hour for a median completion time of 12 min. Due to Prolific's policies, which penalise participants for submissions rejected by researchers (such as for excessive speed, missing data, or failing attention checks), this online platform has demonstrated its ability to ensure high data validity ^87^. To further ensure data quality, we recruited Prolific users with 95–100% approval rate from participation in previous studies, and limited completion time to 30 min. Participants were given the chance to read the study general goal, procedure and methods before signing up and being redirected to the Gorilla experimental platform (www.gorilla.sc), where they provided written consent to participate. The experiment consisted of one task and a series of questionnaires, which were created and hosted using Gorilla Task Builder 2 and Questionnaire Builder 2. The study received ethical approval from the Ethics Committee at the Technische Universität Dresden and was carried out in accordance with the approved guidelines and regulations.

### Task

The study is based on an observational task in which participants are exposed to images of social interactions that include touch. On commencing the experimental task, participants were given the following introductory information:

“In the next screens you will see a series of comics representing everyday interactions between friends living together (two humans or a companion robot and a human). The robot and the human have a friendly relationship and share their daily life. The robot knows and can move around the home environment, engage in joint activities and has communication skills to allow for conversations with the human. Similarly, the human is familiar with the robot and interacts with them on a daily basis.”

They were then presented with pictures of 2 female human characters (called Anna and Sarah) and a humanoid robot (called Pepper). Across 4 trials, they were presented with scenes depicting the two humans (H) or a human and a robot (R) sitting one in front of the other at the table of a living room. In a 2 × 2 design, comics were created by a combination of 2 factors:

- Partner: there was a human interacting with either another human (H) or the robot (R)
- Role: participants were asked questions about how they perceived the one character that was either vulnerable (V) or comforting the other (C).

Moreover, each scene consists of 3 segments: in the first picture one of the characters expresses emotional vulnerability (i.e., says “I am sad”) (V). In the second picture the other character initiates touch by placing their hand on the other’s arm in a comforting manner (C). In the last picture, the receiver reciprocates the touch (Fig. 1). Participants completed 4 trials organized into 2 blocks: human–robot (R) interaction and human–human interaction (H). Each block comprised 2 trials where the roles of characters alternated between being depicted as vulnerable or comforting. To ensure randomization, the order of block presentation was randomized between participants, as well as the order of trials within each block. Thus, although the 2 human–robot trials and the 2 human–human trials were always presented together, their sequence was randomized.

After the presentation of each scene, participants rated the observed social interaction reporting their agreement (on a 7-point Likert scale ranging from 1—strongly agree—to 7—strongly disagree) with statements about the:

- Character’s trustworthiness (Trust questionnaire, adapted from^14^): 9 items that capture participants’ trust in a certain character (highlighted in Fig. 1 through a yellow circle) considering 3 aspects of trust (i.e., integrity, benevolence, ability). As for the H conditions, participants were always asked about Anna’s trustworthiness (the blond female character in Fig. 1), who was either vulnerable or comforting the other human. As for the R conditions, questions referred to Pepper’s trustworthiness, that was either vulnerable or comforting the human. Table S1 of the Supplementary Information reports the adaptation of the trust questionnaire to assess trustworthiness of either the human or robot character. Three scores are calculated summing up the responses to items grouped by subscale. Higher scores indicate higher trustworthiness of the character.

Afterward, participants were sequentially shown again the first (initiation) and second (reciprocity) touch phases of the current scene. For each phase, they reported their agreement (on a 7-point Likert scale ranging from 1—strongly agree—to 7—strongly disagree) with statements about the observed interaction:

- Interaction Realism: 1 item “The interaction was realistic”
- Touch Appropriateness: 1 item “Touch was appropriate”
- Touch Pleasantness: 1 item “Touch was pleasant”

Moreover, they rated the:

- Characters’ affective state by clicking on a point of an EmojiGrid^62^ that best represented how the person(s) in the picture felt. The EmojiGrid is a square grid labelled with emoticons expressing different levels of emotional valence (e.g., sad vs. smiling face) on the x axis and arousal (e.g., sleepy vs. excited face) on the y axis. Participants clicked on a single point inside the grid, which represents the combination of valence and arousal they attribute to the scene displayed.

### Questionnaires

At the end of the task, participants filled out a series of questionnaires about themselves.

The Social Touch Questionnaire (STQ) is a 20-item scale that measures participants’ aversion towards social situations involving touch^88^. On a 5-point Likert scale ranging from 0 (not at all) to 4 (extremely), participants indicate how characteristic or true each statement is of them. A total STQ score is calculated summing up the responses to all items after reversing those that express positive attitudes towards touch (e.g., “I generally like when people express their affection towards me in a physical way”). Higher scores indicate a participant’s dislike for social touch.

The Propensity to trust scale is a 4-item scale that measures individuals’ dispositional trust in other people^89^. On a 7-point Likert scale ranging from 1 (strongly disagree) to 7 (strongly agree), participants express to what extent they agree with statements like “I usually trust people until they give me a reason not to trust them”. A total score is calculated summing up the responses to all items. Higher scores indicate higher propensity to trust other people.

The Propensity to trust technology scale is a 3-item scale that measures individuals’ dispositional trust towards technology in general (Trusting Stance—General Technology^27^). On a 7-point Likert scale ranging from 1 (strongly disagree) to 7 (strongly agree), participants report to what extent they agree with statements like “My typical approach is to trust new technologies until they prove to me that I shouldn’t trust them”. A total score is calculated summing up the responses to all items. Higher scores indicate higher propensity to trust technology.

They also reported their previous experience with robots with 1 item on a 5-point Likert scale from 1 (none) to 5 (a lot): “How much experience have you had with robots?”. An optional open question gave them the possibility to briefly describe such previous experiences with robots.

### Statistical approach

#### Sample size specification

To establish the sample size, we run a priori power analysis (using GPower 3.1) on the main effects of interest. From previous literature, we can expect a main effect size of touch on trust towards a robot to be around Cohen’s d = 0.23^48^. Robot-related characteristics have been found to be moderately associated with trust in human–robot interaction, with r̄ =  + 0.24 (according to a meta-analysis from^19^). Individual (e.g., gender) differences on touch pleasantness ratings previously showed effect sizes around g = 0.25 (according to a meta-analysis from^90^). We therefore run a power analysis for F tests, with repeated measures and within-subjects design, effect size f = 0.115 (conversion from Cohen’s d = 0.23); alpha error probability = 0.05; power = 0.80, 4 conditions (2 Partners * 2 Roles) and 2 measurements (Touch phases) per condition, resulting in a required sample size of n = 152.

### Variables of interest

Below is a description of the variables included in the statistical models. Descriptive statistics (means, standard deviations) of the Dependent Variables (DVs) by relevant experimental conditions are reported in the Supplementary Information.

- *Dependent variables* (DVs—continuous variables from self-reported perceptions of the task stimuli): characters’ Trustworthiness, interaction Realism, touch Appropriateness, touch Pleasantness, characters’ affective state in terms of Valence and Arousal.
- *Independent variables* (IVs—2-level categorical factors representing the experimental conditions): Partner (human vs robot), Role (vulnerable vs comforting), Touch phase (initiation vs reciprocity).
- *Moderators* (self-reports filled out at the end of the task, which are hypothesised to moderate the effect of the IVs on the DVs): Propensity to trust others, Propensity to trust technology.
- *Covariate* (self-report filled out at the end of the task, which is hypothesised to have a direct main effect on the DVs): Touch aversion (total STQ).
- *Control variables*: Gender (female vs male—no participants reported non-binary gender identities), Participant (random effect of individual variability that accounts for the repeated measure, within-subjects design of the experiment).

### Pre-processing

As for the EmojiGrid data, if participants clicked more than once, the last click was considered the definitive answer for analysis (as per the instructions displayed upon presentation of the EmojiGrid). Each response is encoded by coordinates on the x-axis (valence) and y-axis (arousal), which are then analysed separately (as in^62^). Since the size of the participants’ screens varies, the coordinates were normalised by dividing the x-value by the width of each participant's grid size and the y-value by its height. Because of the way the grid is positioned on the Gorilla Task Builder screen, raw values on the x-axis (valence) range from 0 (left) to the maximum (right). On the other hand, the raw values on the y-axis range from the maximum (bottom) to zero (top), and have therefore been reversed. Moreover, the EmojiGrid scale is conceptualised as a matrix where the neutral affective state (namely, the “true” 0,0 position) is located in the centre of the grid. Therefore, we rescaled the normalised response coordinates so that both valence and arousal range from − 50 to + 50. Thus, in our statistical analyses, higher values of valence and arousal indicate greater valence and arousal. Negative values on the valence dimension indicate responses on the left side of the EmojiGrid, and negative values on the arousal dimension indicate responses on the bottom side of the grid.

### Generalised linear mixed-effects models

Statistical analyses have been run with R, version 4.3.0. Generalised linear mixed-effects models were employed to account for the repeated measures design of the experiment (i.e., trials nested within participants, which has been included as a random effect in the analyses). We specified the research hypotheses on the link between each dependent variable and the predictors of interest as statistical models. Analysis of deviance (Type III Wald chi-square test, r package ‘*car’*^91^) was used for assessing the effect of individual predictors and interactions included in the models. As an index of goodness of prediction, conditional R2 (the ratio of variance explained by fixed and random effects over total variance) and marginal R2 (the ratio of variance explained by fixed effects over total variance) were calculated to quantify the variance explained by the whole model (including the contribution of individual variability) or the fixed effects only (excluding the contribution of individual variability)^92^. Higher percentages of explained variance indicate a stronger association between the dependent variable and the predictors, with the model making better predictions.

### Model specification

#### Trustworthiness

With a generalised linear mixed effect model, we tested how perceived trustworthiness was predicted by the 2-way interaction between Partner (Human or Robot) and Role (Comforting or Vulnerable), including touch aversion as a covariate, controlling for gender and individual variability. Moreover, the model tested whether the effect of Partner was moderated by individuals’ propensity to trust others and technology, and the subscales of the trustworthiness measure (ability, benevolence, integrity). Below, the formula is reported.

$$\begin{aligned} Trustworthiness\sim & Partner*Role + Partner*{\text{Propensity to trust others}} + \, Partner \, *{\text{Propensity to trust technology}} \\ & + \, Partner*TrustSubscale + Touch \, aversion + Gender + (1|Id) \\ \end{aligned}$$

### Interaction

With several generalised linear mixed effect models, we tested how each DV (interaction realism, touch appropriateness, touch pleasantness, valence, arousal) was predicted by the 2-way interactions between Partner and Role, and Partner and touch phase (initiation or reciprocity), controlling for gender and individual variability. Moreover, the model tested whether the effect of Partner was moderated by individuals’ propensity to trust others and technology, and whether the DV was covarying with the individuals’ touch aversion. Below, the formula is reported.

$$\begin{aligned} DV\sim & Partner*Role + Partner*Touch\_phase + Partner*Propensity \, to \, trust \, others \\ & + Partner*Propensity \, to \, trust \, technology + Touch \, aversion + Gender + \left( {1|id} \right) \\ \end{aligned}$$

### Informed consent

Informed consent was obtained from all subjects and/or their legal guardian(s).

### Supplementary Information

Supplementary Information.

## Data Availability

The original dataset and analysis script are available from the OSF public repository at the following URL: https://osf.io/7bf2m/?view_only=b2c801bfbc4d4467b461a22c492dac31
