# Author Correction: Propensity to trust shapes perceptions of comforting touch between trustworthy human and robot partners

**DOI:** 10.1038/s41598-024-60639-w

**Published:** 2024-05-03

**Authors:** Irene Valori, Yichen Fan, Merel M. Jung, Merle T. Fairhurst

**Affiliations:** 1 Chair of Acoustics and Haptics, Technische Universität Dresden, Dresden, Germany; 2 Centre for Tactile Internet with Human-in-the-Loop (CeTI), Technische Universität Dresden, Dresden, Germany; 3 Chair of Industrial Design Engineering, Technische Universität Dresden, Dresden, Germany; 4 6G-Life, Dresden, Germany; 5 Department of Cognitive Science and Artificial Intelligence, Tilburg University, Tilburg, The Netherlands

Correction to: *Scientific Reports* 10.1038/s41598-024-57582-1, published online 21 March 2024

The original version of this Article contained errors in the legend of Figure 4.

**“How does it feel?** Significant effects predicted by Valence and Arousal models; n_participants_ = 142; n_observations_ = 1115. Panel (**A**) visualises participants’ clicks on the EmojiGrid, averaged by experimental condition (scenario) and touch phase. Results suggest that the characters of the observed interaction are perceived to be in a less negative affective state when touch is reciprocated by the receiver. That effect is moderated by the Partner factor, with the reciprocity effect being smaller in the Robot condition (**B**). A more aroused state is reported when touch happens between a human and the robot rather than two humans. More specifically, perceived arousal is higher when the robot is the one expressing vulnerability (**C**). Arousal also increases when touch is reciprocated by the receiver, with no significant difference between human and robot partner (**D**). Individuals’ propensity to trust others is positively associated with perceived arousal, only in human-to-human interactions (**E**). Individuals’ touch aversion is associated with reduced arousal.”

now reads:

“**How does it feel?** Significant effects predicted by Valence and Arousal models; n_participants_ = 142; n_observations_ = 1115. Panel (**A**) visualises participants’ clicks on the EmojiGrid, averaged by experimental condition (scenario) and touch phase. Results suggest that the characters of the observed interaction are perceived to be in a less negative affective state when touch is reciprocated by the receiver. That effect is moderated by the Partner factor, with the reciprocity effect being smaller in the Robot condition (**B**). A more neutral arousal state (closer to 0, which represents the center of the EmojiGrid) is reported when touch happens between a human and the robot rather than two humans. This is especially evident when the robot is the one expressing vulnerability (**C**). Arousal also becomes more neutral when touch is reciprocated by the receiver, with no significant difference between human and robot partner (**D**). Individuals’ propensity to trust others is associated with more neutral perceived arousal, only in human-to-human interactions (**E**). Individuals’ touch aversion is associated with more neutral arousal (**F**).”

The original Article has been corrected.

