# Supplementary material for: Propensity to trust shapes perceptions of comforting touch between trustworthy human and robot partners
Source: Sci Rep. 2024 Mar 21;14:6747. doi: 10.1038/s41598-024-57582-1 (PMC10957953; doi:10.1038/s41598-024-57582-1)
Supplement: Supplementary file 1 — Supplementary Information. [file 41598_2024_57582_MOESM1_ESM.pdf]

Propensity to trust shapes perceptions of comforting touch between trustworthy human and robot partners

Irene Valori [1,2]*, Yichen Fan [3,4], Merel M. Jung [5], Merle T. Fairhurst [1,2,4]

[1] Chair of Acoustics and Haptics, Technische Universität Dresden, Dresden, Germany

[2] Centre for Tactile Internet with Human-in-the-Loop (CeTI), Technische Universität Dresden, Dresden, Germany

[3] Chair of Industrial Design Engineering, Technische Universität Dresden, Dresden, Germany

[4] 6G-Life

[5] Department of Cognitive Science and Artificial Intelligence, Tilburg University, Tilburg, the Netherlands

*Corresponding author: irene.valori@tu-dresden.de

Supplementary Information

Table S1: Character's trustworthiness (Trust questionnaire, adapted from Gefen, 2002)

Subscale	Human	Robot
Integrity	1. Promises made by Anna are likely to be reliable<br>2. I do not doubt the honesty of Anna<br>3. I expect Anna will keep promises they make	1. Promises made by Pepper are likely to be reliable<br>2. I do not doubt the honesty of Pepper<br>3. I expect that Pepper will keep promises they make
Benevolence	4. I expect Anna has good intentions toward Sarah<br>5. I expect Anna's intentions are benevolent<br>6. I expect Anna is well meaning	4. I expect that Pepper has good intentions toward Anna<br>5. I expect that Pepper's intentions are benevolent<br>6. I expect that Pepper is well meaning
Ability	7. Anna understands the environment they live in<br>8. Anna knows about Sarah<br>9. Anna knows how to provide support to Sarah	7. Pepper understands the environment they live in<br>8. Pepper knows about Anna/Sarah<br>9. Pepper knows how to provide support to Anna/Sarah

Qualitative data: previous experience with robots

At the end of the experiment, participants were given the possibility to briefly describe their previous experience with robots, if any. Only 13 participants described interactions with social types of robots. These descriptions are listed in full below.

1. I had an experience with a teller, the experience made it easier and efficient.
2. 1. Robot as a waiter in a Japanese restaurant. It was at the beginning weird and a bit scary but in the end, I liked the usefulness and the fastness of the service it provided. 2. Robot as a secretary in a luxury hotel. It was not the best experience ever, it did not understand half of the requests and in the end, I called a person.
3. I went to a museum in Amsterdam where they had the Tokyo experience. The same robot as shown in this experiment was used at a display where you could interact with Pepper.

4. Only once, in a hotel But it was an interaction that lasted less than a minute. The robot guided us to the reception, and it was nice, although I don't think you can trust robots that much, at least not now. Technology keeps moving forward and, just like any software, updates can be part of the success of robots. People need to interact with them more and more so that programmers learn how to make robots more and more reliable.
5. Robots in cafes that bring you your orders.
6. The only experience I had was a waiter robot in a hotel who basically drove around with food on it and you could pick it up if you were in the mood for having that food.
7. I interacted with robots at restaurants. They were delivering the food. I enjoyed the experience, it was different from what I was used to.
8. It was a robot at a hotel that served me breakfast. I lived (*loved*?) interacting less with human beings. It just greeted and put food on the table without initiating unnecessary small conversation.
9. They delivered the food to the table at a restaurant, it was very brief but they delivered it well. Little unpersonal in my opinion.
10. I've recently had an experience with a store robot that helped me find what I was looking for.
11. A robot waiter in a restaurant. He delivered the dishes to the tables. I found it innovative, unusual, exciting and fun.
12. I only had experience with robots at restaurants but had no control over them; I only saw them working.
13. I studied electrical engineering so I've made robots (like one that could follow a line/route for example) I've been to expo's where social robots were present although I didn't interact with them directly and I've read and seen stuff about social robots online both from a technical point of view as well as surface level. In general I love the technical side of a social robot but actually interacting with one makes me slightly uncomfortable/doesn't interest me much.

From these qualitative data, we appreciate how rare, brief and limited interactions with social robots are. Even in the case of experts, such as participant 14 who qualifies as an engineer dealing with robots for work, interest in social interactions with robots appears to be limited to curiosity and enjoyment of something new, but no motivation for more complex and long-term interactions emerges.

Descriptive statistics

Mean values and Standard Deviations (SD) of all dependent variables by subscale (trustworthiness) and experimental condition are reported in the Table S2 below.

Table S2: Descriptive statistics

		Scenario			
		H_C	H_V	R_C	R_V
	**Subscale**				
Trustworthiness	Ability	5.7 (1.1)	5.6 (1.1)	4.4 (1.6)	4.1 (1.5)
	Benevolence	5.4 (1.1)	5.3 (1.1)	4.7 (1.5)	4.3 (1.4)
	Integrity	5.0 (1.2)	5.0 (1.2)	4.5 (1.4)	4.1 (1.5)
	**Touch phase**				
Interaction realism	Initiation	5.6 (1.3)	5.6 (1.4)	4.1 (1.7)	3.4 (1.8)
	Reciprocity	5.7 (1.3)	5.6 (1.4)	4.1 (1.7)	3.6 (1.8)
Touch appropriateness	Initiation	5.9 (1.1)	5.8 (1.2)	4.9 (1.6)	4.3 (1.8)
	Reciprocity	5.9 (1.2)	5.9 (1.2)	4.8 (1.7)	4.3 (1.8)
Touch pleasantness	Initiation	5.7 (1.3)	5.7 (1.3)	4.5 (1.7)	3.9 (1.8)
	Reciprocity	5.8 (1.3)	5.7 (1.4)	4.4 (1.7)	4.0 (1.8)
Valence	Initiation	-18.7 (24.3)	-15.1 (24.5)	-15.6 (20.1)	-8.3 (18.7)
	Reciprocity	-0.2 (27.3)	-2.4 (26.8)	-1.9 (23.5)	-1.7 (20.6)
Arousal	Initiation	-27.4 (19.8)	-27.3 (19.5)	-24.5 (18.8)	-18.4 (20.3)
	Reciprocity	-22.7 (21.5)	-23.1 (21.7)	-22.1 (19.9)	-17.6 (20.5)

Visualisation of statical results

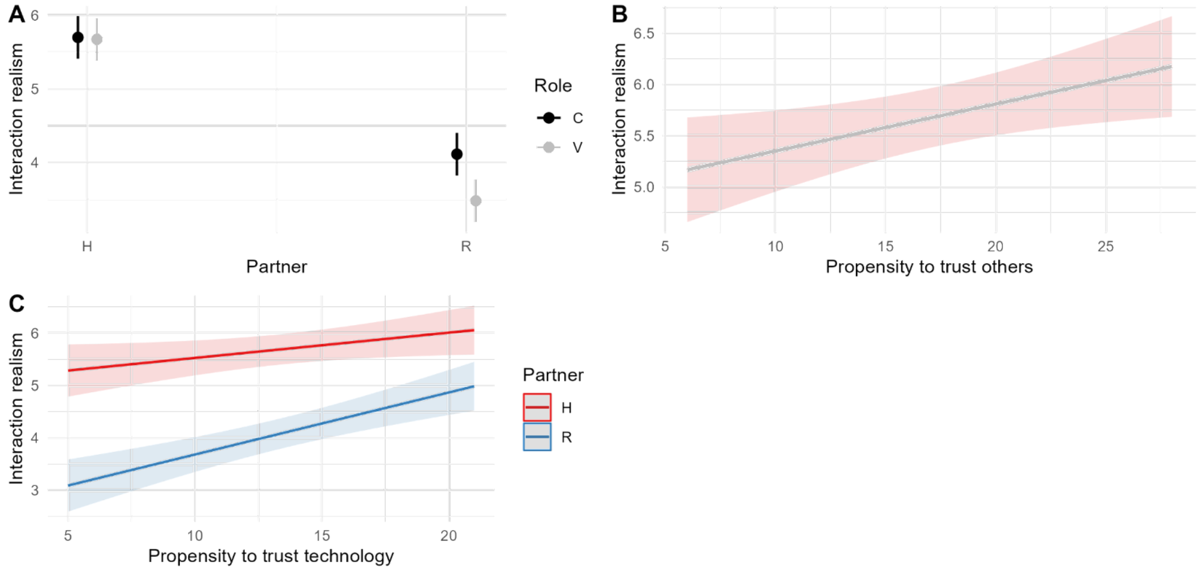

Fig S1: Interaction realism: significant effects predicted by the model. $n_{participants}$=152; $n_{observations}$=1216. Human-to-human interactions are perceived as more realistic than human-robot interactions. The latter is less realistic, especially when the robot expresses vulnerability (A). Individual differences among participants moderate these effects. In general, social interactions involving comforting touch are perceived as more realistic by those who trust others more (B). The propensity to trust technology is linked to perceptions of human-robot interactions as more realistic (C).

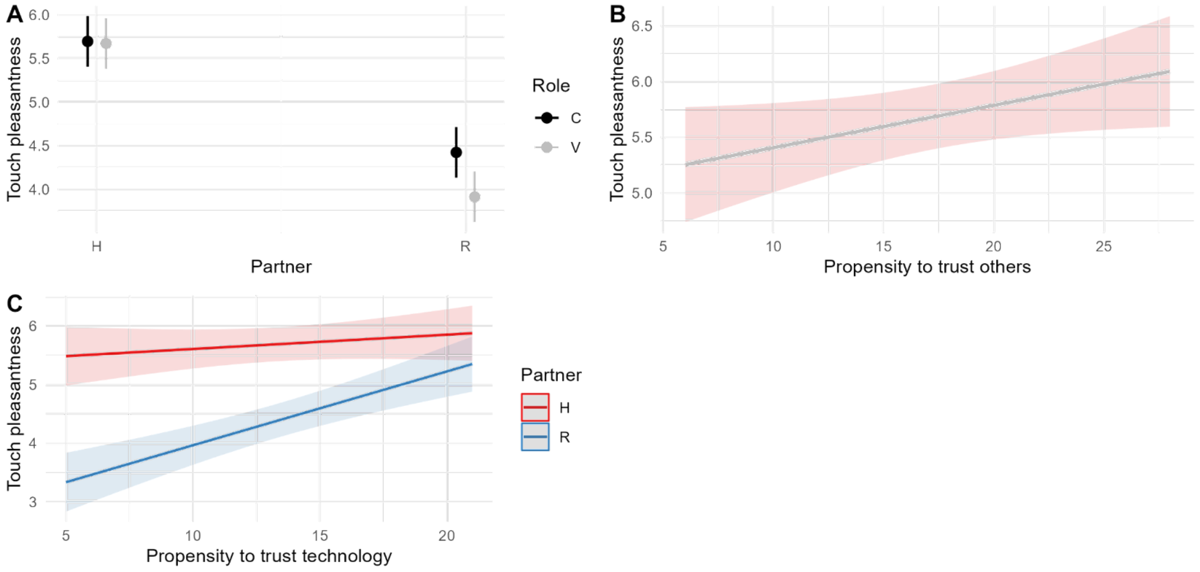

Fig S2: Touch pleasantness: significant effects predicted by the model. $n_{participants}$=152; $n_{observations}$=1216. Human-to-human comforting touch is perceived as more pleasant than human-robot touch. The latter is less pleasant, especially when the robot expresses vulnerability (A). Individuals' propensity to trust others is associated with increased pleasantness (B). Moreover, individuals' propensity to trust technology is associated with increased perception of robot touch as pleasant, thus reducing the gap between humans and robots (C).
